# Paeonol Attenuates Quorum-Sensing Regulated Virulence and Biofilm Formation in *Pseudomonas aeruginosa*

**DOI:** 10.3389/fmicb.2021.692474

**Published:** 2021-08-04

**Authors:** Dan Yang, Suqi Hao, Ling Zhao, Fei Shi, Gang Ye, Yuanfeng Zou, Xu Song, Lixia Li, Zhongqiong Yin, Xiaoli He, Shiling Feng, Helin Chen, Yu Zhang, Yuanze Gao, Yinglun Li, Huaqiao Tang

**Affiliations:** ^1^College of Veterinary Medicine, Sichuan Agricultural University, Chengdu, China; ^2^College of Science, Sichuan Agricultural University, Chengdu, China; ^3^College of Life Science, Sichuan Agricultural University, Yaan, China

**Keywords:** paeonol, quorum sensing, *Pseudomonas aeruginosa*, anti-virulence, biofilm

## Abstract

With the prevalence of multidrug-resistant bacteria and clinical -acquired pathogenic infections, the development of quorum-sensing (QS) interfering agents is one of the most potential strategies to combat bacterial infections and antibiotic resistance. Chinese herbal medicines constitute a valuable bank of resources for the identification of QS inhibitors. Accordingly, in this research, some compounds were tested for QS inhibition using indicator strains. Paeonol is a phenolic compound, which can effectively reduce the production of violacein without affecting its growth in *Chromobacterium violaceum* ATCC 12472, indicating its excellent anti-QS activity. This study assessed the anti-biofilm activity of paeonol against Gram-negative pathogens and investigated the effect of paeonol on QS-regulated virulence factors in *Pseudomonas aeruginosa*. A *Caenorhabditis elegans* infection model was used to explore the anti-infection ability of paeonol *in vivo*. Paeonol exhibited an effective anti-biofilm activity against Gram-negative bacteria. The ability of paeonol to interfere with the AHL-mediated quorum sensing systems of *P. aeruginosa* was determined, found that it could attenuate biofilm formation, and synthesis of pyocyanin, protease, elastase, motility, and AHL signaling molecule in a concentration- and time-dependent manner. Moreover, paeonol could significantly downregulate the transcription level of the QS-related genes of *P. aeruginosa* including *lasI/R, rhlI/R, pqs/mvfR*, as well as mediated its virulence factors, *lasA, lasB, rhlA, rhlC, phzA, phzM, phzH*, and *phzS*. *In vivo* studies revealed that paeonol could reduce the pathogenicity of *P. aeruginosa* and enhance the survival rate of *C. elegans*, showing a moderate protective effect on *C. elegans*. Collectively, these findings suggest that paeonol attenuates bacterial virulence and infection of *P. aeruginosa* and that further research elucidating the anti-QS mechanism of this compound *in vivo* is warranted.

## Introduction

Quorum sensing (QS) is a type of density-dependent cell-to-cell communication mechanism used by bacteria to detect community density. QS coordinates its clustering behavior by producing and sensing signaling molecules called autoinducers (AIS) and their receptors (Swift et al., 1996; Davies et al., 1998). Three different types of AIs have been discovered: *N*-acyl L-homoserine lactone (AHLs) exploited by Gram-negative species, small peptide signals exploited by Gram-positive species and autoinducer-2 for inter-species communication (Ng and Bassler, 2009). Once the bacterial population density reaches a critical threshold, the QS system activates the expression of virulence-associated genes to control a vast set of relevant phenotypes including biofilm-formation, motility, exopolysaccharide production, virulence factors, motility, and bioluminescence (Bassler et al., 1993; Miller and Bassler, 2001).

The excessive and indiscriminate use of antibiotics has eventually led to the emergence of multidrug-resistant bacteria that are difficult to control using conventional antibiotics, as most evident in the ESKAPE-pathogens (*Enterococcus faecium*, *Staphylococcus aureus*, *Klebsiella pneumoniae*, *Acinetobacter baumannii*, *Pseudomonas aeruginosa*, and *Enterobacter* spp.; Tacconelli et al., 2002; Boucher et al., 2009; MacPherson et al., 2009). Biofilm is a barrier system, which can protect the bacteria from the immune reaction of the host, prevent the entry of disinfectants and antibiotics, make the bacteria develop multi-drug resistance, and lead to clinical infection which is difficult to cure (Dunne, 2002). About 80% of all microbial-induced infections are biofilm-based. Quorum sensing has known to play a crucial role in biofilm formation of many pathogenic bacteria, serving as biofilm quorum-sensing molecules (Fuqua and Greenberg, 2002). *Pseudomonas aeruginosa* (*P. aeruginosa*) is a pervasive Gram-negative organism and an opportunistic pathogen in humans capable of causing multiple diseases including chronic panbronchiolitis, cystic fibrosis (CF), and chronic urinary tract infections (Lyczak et al., 2002; Hassett et al., 2009). The QS networks in *P. aeruginosa* comprise four distinct interwoven circuits, namely *las*, *rhl*, *pqs*, and *iqs*, with the *las* system positively at the upstream position regulating both the *rhl* and *pqs* systems, which also cross-regulate in a complex manner (Schuster and Greenberg, 2006; Welsh et al., 2015; Huang et al., 2019). The QS system is essential for the production of different signaling molecules including *P. aeruginosa* autoinducer 1 [*N*-(3-oxododecanoyl-homoserine lactones) or 3-oxo-C12-HSL], *Pseudomonas* autoinducer 2 (*N*-butyryl-homoserine lactones or C4-HSL), and PQS (2-heptyl-3-hydroxy-4-quinolone), each of which acts as an autoinducer of a specific sensing and responding system (Williams and Camara, 2009; Ismail et al., 2016). Once these signals reach a threshold, the collective behavior changes by activating the sensor or modulating the protein (Ilangovan et al., 2013). The QS system in *P. aeruginosa* regulates biofilm formation, motility, and the expression of virulence-associated factors such as pyocyanin, proteolytic enzymes, extracellular proteases, and rhamnolipids (Parsek and Greenberg, 2000). Therefore, *P. aeruginosa* has become the typical model organism for assessment of antibacterial, anti-QS and anti-biofilm activity.

Given the essential role of QS in bacterial pathogenicity and virulence, it is considered a novel and potential therapeutic strategy for coping with bacterial infections and antibiotic resistance (Packiavathy et al., 2014; Hernando-Amado et al., 2019). Natural products obtained from plants, microorganisms, and marine organisms have shown promise in inhibiting QS signaling and biofilm formation rather than targeting bacterial growth (Valdez et al., 2005; Khan et al., 2009; Glamoclija et al., 2015). *Chromobacterium violaceum* is a QS biosensor strain that produces the pigment, violacein, in response to QS-related gene expression, and is, thus, widely used to research QS activities (Singh et al., 2009). Paeonol is one of the main compounds present in traditional Chinese medicine, including Moutan cortex, *Cynanchum paniculatum*, and *Paeonia lactiflora* Pal. Paeonol is known to exert multiple pharmacological effects, immunomodulation, and liver and kidney protection, antitumor, antipyretic, analgesic, anti-inflammatory, and antibacterial and has been widely used as a nutrient supplement (Zhang et al., 2019; Tsai et al., 2020).

However, to the best of our knowledge, there are no studies showing the anti-biofilm and anti-QS activity of paeonol. Thus, in this study, we preliminary evaluated the effect of paeonol on bacterial biofilm formation. Further, we investigated the mechanism of paeonol *in vitro* against *P. aeruginosa*-based anti-QS by detecting the virulence phenotypes, biofilm-formation, and related gene expression. The protective effect of paeonol on the pathogenicity of *P. aeruginosa* was determined *in vivo* using a nematode model.

## Materials and Methods

### Bacterial Strains, Chemicals, and Culture Conditions

*Pseudomonas aeruginosa* (PAO1, ATCC 27853, and ATCC 9027), *P. aeruginosa* clinically isolated stains (PAO3401, isolated from Chinchilla and identified in the laboratory), *C. violaceum* (ATCC 12472 and CV026), *A. baumannii* 17978, *Escherichia coli* (ATCC 25922 and OP50), *Salmonella typhimurium* 14028 and MRSA TCH1516 that were stored in our laboratory were used in this study. The cryopreserved *P. aeruginosa* (PAO1, ATCC 27853, ATCC 9027, and PAO3401), *A. baumannii* 17978, *E. coli* (ATCC 25922 and OP50), *S. typhimurium* 14028 and MRSA TCH1516 were cultured overnight in Luria-Bertani (LB) broth in a rotator shaker with agitation at 180 rpm at 37°C. *C. violaceum* (ATCC 12472 and CV026) were cultured overnight at 30°C aerobically in LB broth in a rotator shaker with agitation 180 rpm. The test compounds were dissolved in dimethyl sulfoxide (DMSO), including protocatechuic aldehyde, aniseed aldehyde, paeonol, carvacrol, polydatin, saikosaponin A, atractylenolide, gallate, calycosin-7-glucoside, veratric acid, myristic acid, ellagic acid, glycyrrhiza acid, aurantiamarin, baicalin, rutin, puerarin, and sophocarpidine (Yuanye Bio-Technology Co., Shanghai, China). DMSO and fresh LB broth were used as controls. The chemicals and reagents used in this study was analytical grade. For each experiment, the bacteria were cultured overnight and re-inoculated at 1:1,000 dilution (approximately 5 × 10^5^ CFU/mL) in fresh LB broth. Cell growth was determined based on the optical density that was measured at a wavelength of 600 nm using microplate reader (Thermo Fisher Scientific, Waltham, MA, United States). Then, *C. elegans* N2 cultures and *E. coli* OP50 were maintained on nematode growth medium (NGM) as a food source at 20°C. IPEC-J2 cells were cultured in DMEM medium (Gibco, Grand Island, New York, United States) supplemented with 10% fetal bovine serum (Gibco, Oshima, New York, United States) and 1% penicillin/chain (Solarbio, Beijing, China) at 37°C with 5% CO_2_ in the incubator.

### Preliminary Screening of AI-1 Quorum Sensing Inhibitors

A quantitative assay for violacein was carried out as previously described with few modifications (Blosser and Gray, 2000). Briefly, 200 μL of *C. violaceum* ATCC 12472 (approximately 5 × 10^5^ CFU/mL) was incubated individually with different concentrations of chemical compounds at 30°C for 18 h and OD_600_ was measured by microplate reader. After incubation, the violacein from 200 μL of the bacterial culture was extracted using 100 μL of 10% SDS and 400 μL of *n*-butanol. After centrifugation at 8,900×*g* for 5 min, 200 μL the organic layer was taken in a 96-well plate. The absorbance was determined at 595 nm to quantify the violacein in the supernatant.

*Chromobacterium violaceum* ATCC 12472 was cultured in LB medium at 30°C with shaking at 180 rpm, diluted in a 1:100 ratio with LB solid medium, and poured into Petri dishes (McLean et al., 2004). Then, 20 μL of the test compounds at a concentration of 20 mg/mL were added to the wells of the plates and incubated at 30°C for 24 h. Due to a loss of violacein pigmentation, the anti-QS activity was evaluated by the production of a colorless and opaque but turbid halo around the well, and was measured from the outer edge of the disk to the edge of the anti-QS inhibition zone.

### Minimum Inhibitory Concentration Assay and Growth Curves

Various concentrations of paeonol were examined against the selected bacteria strains and their inhibitory activity was determined using a modified broth microdilution method as stated in the Clinical and Laboratory Standards Institute (2012). Twofold dilutions of paeonol were prepared using LB broth in a 96-well plate with final concentrations ranging from 16 to 2,048 μg/mL. A suspension of bacteria strains at a final test concentration of about 5 × 10^5^ CFU/mL was added to each well and incubated at 37°C for 24 h. The MIC was defined as the minimum concentration of paeonol that inhibited the growth of the selected microorganisms (Clinical and Laboratory Standards Institute, 2012).

A growth-curve analysis was performed for the PAO1 strain to determine the impact of the sub-inhibitory concentrations of paeonol on growth (Jorgensen, 1993). Overnight grown cultures of PAO1 were prepared using LB broth and supplemented with paeonol (0, 128, 256, and 512 μg/mL). LB medium and the same concentration of DMSO were used as negative controls. The conical flask was cultured in a rotator shaker while being agitated at 180 rpm and 37°C. The optical density was determined at 600 nm every hour for up to 36 h.

### Anti-biofilm Activity Assay of Paeonol

#### Anti-biofilm Formation Quantitative Assay

For observing the inhibitory effect of paeonol on bacterial biofilm, we incubated the bacterial suspension of *C. violaceum* 12472, *P. aeruginosa* (PAO1, ATCC 27853, ATCC 9027, and PAO3401), *A. baumannii* 17978, *E. coli* 25922, *S. typhimurium* 14028, and MRSA TCH1516. The biofilm-forming ability of selected bacteria was determined using the crystal violet method (Lee et al., 2011). Briefly, 200 μL of the bacteria culture (approximately 5 × 10^5^ CFU/mL) was taken in a 96-well plate and incubated with different concentrations of paeonol at 37°C for 24 and 36 h. After incubation, the biofilm was washed with sterile phosphate-buffered saline (PBS) for three times and fixed with 200 μL of methanol (99%) for 15 min. The supernatant was discarded, and the biofilm was stained with 200 μL of 0.1% crystal violet and dissolved in 33% acetic acid for 15 min. The absorbance was measured at 595 nm to quantify biofilm formation.

#### Microscopy Analysis of Paeonol Against *P. aeruginosa*

The method of biofilm formation was conducted as described previously (Murphy et al., 2014). Briefly, biofilms were cultured for 36 h on a glass coverslip using 24 well plates with paeonol (0–512 μg/mL) treated and untreated cultures of PAO1. The total biofilm biomass was determined by washing the glass coverslip with PBS three times, drying at 60°C, staining with 0.1% crystal violet, and then observing under the light microscope. Scanning electron microscopy (SEM) was further applied to visualize biofilm architecture. The coverslips were washed for non-adhered bacteria three with PBS and fixed overnight at 4°C in 2.5% (v/v) glutaraldehyde, then processed for SEM using standard protocol.

### Flagellar Motility Assay of Paeonol Against *P. aeruginosa*

Bacterial motility was assayed on agar plates after treatment with different concentrations of paeonol (0, 128, 256, and 512 μg/mL). *P. aeruginosa* (PAO1, ATCC 27853, ATCC 9027, and PAO3401) were cultured in LB broth overnight at 37°C at constant shaking at 180 rpm. Next, 1 μL of *P. aeruginosa* cells was added to the center of the swarming solid medium (1.0% peptone, 0.5% sodium chloride, 0.5% glucose, and 0.5% agar) and swimming soft medium (1.0% peptone, 0.5% sodium chloride, and 0.3% agar). The plates were inoculated at 37°C for 24 h, and the flagellar motility was determined by measuring the diameter (cm) of the traveled cells on the surface of the agar plate (Rashid and Kornberg, 2000).

### Analysis Virulence Phenotypes of *P. aeruginosa*

#### Pyocyanin Quantitative Assay

Pyocyanin production was determined using different concentrations of paeonol in PB medium (2% tryptone, 1% K_2_SO_4_, and 0.14% MgCl_2_) by the chloroform-HCl extraction method (Chong et al., 2011). A volume of 2 mL of the PAO1 culture (approximately 5 × 10^5^ CFU/mL) was incubated with different concentrations of paeonol (0–512 μg/mL) at 37°C for 48 h in a 24-well plate. After centrifugation at 10,000×*g* for 10 min, the supernatant of the PAO1 culture was extracted with 0.75 mL of chloroform and 0.2 M HCl (0.25 mL) was added to the chloroform layer. After centrifugation, the HCl was removed and pyocyanin was quantified at 520 nm.

#### Proteolytic Activity Assay

A protease quantitative analysis was performed according to a previously published method (Hentzer et al., 2002) with minor modifications. Briefly, 2 mL of PAO1 culture (approximately 5 × 10^5^ CFU/mL) was incubated with different concentrations of paeonol (0–512 μg/mL) at 37°C for 24 h. After centrifugation, the filter-sterilized supernatant (150 μL each) of PAO1 treated with paeonol was added with 250 μL of 2% azocasein (w/v)-*Tris*/HCl solution (pH 7.8). Next, the azocasein-treated solutions were incubated at 4°C for 4 h and the reaction was terminated by the addition of trichloroacetic acid (10%, 1.2 mL) for 15 min. After centrifugation at 10,000×*g* for 10 min, the supernatant was added to 1 M NaOH (1.4 mL). Protease activity was determined by measuring the absorbance at a wavelength of 440 nm.

#### Elastase Activity Assay

The elastasse qualitative analysis was monitored according to previously reported methods (Adonizio et al., 2008). Briefly, 2 mL of PAO1 cultures (approximately 5 × 10^5^ CFU/mL) were incubated with different concentrations of paeonol (0–512 μg/mL) at 37°C for 24 h. The cultures were centrifuged for 10 min at 10,000 rpm and filter-purified by a 0.22 μm nylon filter, 100 μL supernatant were added to 400 μl of ECR buffer (100 mM *Tris*, 1 mM CaCl_2_, pH 7.2) containing 20 mg ECR. Then, the mixture was incubated at 37°C for 16 h with shaking (200 rpm). The insoluble ECR was removed by centrifugation, and the absorbance of the supernatant at 495 nm was measured to evaluate elastase activity.

#### Rhamnolipids Quantitative Assay

Rhamnolipids of *P. aeruginosa* were cultured in PPGAS medium (20 mM KCl, 20 mM NH_4_Cl, 0.5% glucose, 1.6 mM MgSO_4_, 120 mM *Tris*–HCl, and 1.0% peptone) according to a previously published method (Grosso-Becerra et al., 2016). Briefly, 2 mL of the PAO1 cells (approximately 5 × 10^5^ CFU/mL) were allowed to grow in the presence and absence of paeonol at 37°C for 24 h. After centrifugation, the supernatants were extracted twice with ethyl acetate; the organic layers were combined and evaporated to dryness overnight at 50°C. The solid product was dissolved in 500 μL of sterile distilled water. Next, 100 μL of the sample was added to 0.19% orcinol in 53% concentrated sulfuric acid (900 μL) and incubated in a water bath at 80°C for 30 min. After cooling to room temperature (18–25°C), the absorbance of the samples was measured spectrophotometrically at 421 nm.

### AHL Synthesis Activity Assay of Paeonol Against *P. aeruginosa*

The levels of AHL in the PAO1 supernatants were measured after treatment with paeonol by assessing the violacein production by CV026. About 2 mL of the PAO1 culture (approximately 5 × 10^5^ CFU/mL) was incubated with different concentrations of paeonol (0–512 μg/mL) at 37°C for 24 h. After centrifugation, the supernatants were extracted twice with ethyl acetate. The organic layers were combined and evaporated to dryness overnight at 40°C. The yellow precipitate was resuspended in 200 μL of DMSO, and 20 μL was added into the wells of the fresh agar plates containing CV026. The plates were inoculated at 30°C for 24 h. The production of AHL was monitored by measuring the zone of clearance around each well (Ravn et al., 2001).

### Expression of QS Genes on *P. aeruginosa*

Fluorescence real-time PCR was used to detect the expression of the QS genes of PAO1 in the presence of paeonol (0, 128, 256, and 512 μg/mL). Table 1 shows the primers that were used to amplify *lasI, lasR, rhlI, rhlR, pqsA, pqsR, lasA, lasB, rhlA, rhlC, phzm, phzM, phzH, phzS, and pvdQ* (reference gene). Total RNA was then extracted using TRIzol Reagent (Songon Biotech, Shanghai, China) according to the manufacturer’s instructions. Next, the samples were reverse-transcribed to single-stranded cDNA in a 20-μL volume of the reaction mixture using Maxima H Minus First Strand cDNA Synthesis kit (Thermo Fisher Scientific, Waltham, MA, United States). Then, real-time PCR was performed in a 10-μL reaction volume, containing 5 μL of PerfectStartTM Green qPCR SuperMix (TransGen Biotech, Beijing, China), 2 μL of the template cDNA, 1 μL of primers (Shenzhen Huada Gene Research Institute, Shenzhen, China), and 2 μL of DNase/RNase-free water (Tiangen Biotech, Beijing, China). The pvdQ gene was chosen as a reference gene and used to normalize the quantitative PCR data and calculate the relative differences in mRNA expression using the 2^–△△CT^ method.

**TABLE 1:** PCR primers of *Pseudomonas aeruginosa* for analysis of gene expression.

Genes	Primer sequences (5′–3′)	
*lasI*	F: CGCACATCTGGGAACTCA	R: CGGCACGGATCATCATCT
*lasR*	F: CTGTGGATGCTCAAGGACTAC	R: AACTGGTCTTGCCGATGG
*rhlI*	F: GTAGCGGGTTTGCGGATG	R: CGGCATCAGGTCTTCATCG
*rhlR*	F: GCCAGCGTCTTGTTCGG	R: CGGTCTGCCTGAGCCATC
*pqsA*	F: GACCGGCTGTATTCGATTC	R: GCTGAACCAGGGAAAGAAC
*pqsR*	F: CTGATCTGCCGGTAATTGG	R: ATCGACGAGGAACTGAAGA
*lasA*	F: CTGTGGATGCTCAAGGACTAC	R: AACTGGTCTTGCCGATGG
*lasB*	F: AACCGTGCGTTCTACCTGTT	R: CGGTCCAGTAGTAGCGGTTG
*rhlA*	F: TGGCCGAACATTTCAACGT	R: GATTTCCACCTCGTCGTCCTT
*rhlC*	F: GCCATCCATCTCGACGGAC	R: CGCAGGCTGTATTCGGTG
*phzM*	F: ACGGCTGTGGCGGTTTA	R: CCGTGACCGTCGCATT
*phzA*	F: AACGGTCAGCGGTACAGGGAAC	R: AACGGTCAGCGGTACAGG GAAAC
*phzH*	F: GCTCATCGACAATGCCGAACT	R: GCGGATCTCGCCGAACATCAG
*phzS*	F: CCGAAGGCAAGTCGCTGGTGA	R: GGTCCCAGTCGGCGAAGAACG
*pvdQ*	F: GCCGAGGAGATCGTCACC	R: CAGGCGTAGAAGATGTCGGA

### Anti-infection Activity of Paeonol on *C. elegans*

The *C. elegans* population was synchronized according to a method reported previously (Tan et al., 1999b), with some modifications. Briefly, 200 μL of the broth culture of *P. aeruginosa* was spread evenly on NGM agar plates containing paeonol (0, 128, and 256 μg/mL) and cultured for 18 h at 37°C to create a bacterial lawn. Plates coated with *E. coli* OP50 were used as negative controls. Then, 40 synchronized nematodes (L4 stage) of the wild type *C. elegans* N2 strain were selected and suspended onto the plates and counted every 24 h using a stereomicroscope. In the experiment, the nematodes were considered dead when immobilized. The number of surviving nematodes was tabulated and a survival curve was constructed.

### Cell Proliferation Assay

The CCK-8 assay was performed to determine the number of viable cells according to the manufacturer’s protocol. IPEC-J2 cells in logarithmic growth were seeded in 96-well plates (1 × 10^5^ per well) and cultured in DMEM medium supplemented with 10% FBS for 12 h, and then treated with a series of concentrations of paeonol (0, 16, 32, 64,128, 256, 512, and 1,024 μg/ml) for 24 h. Then, 10 μl CCK-8 solution was added to each well of the plates. After incubation at 37°C for another 3 h, the absorbance at 450 nm was detected to determine the number of viable cells.

### Statistical Analysis

All data were processed using SPSS 22.0 (IBM Corporation, Armonk, NY, United States) and are presented as mean ± SD. Graphs were constructed using GraphPad Prism 8.0.1 (GraphPad Software, La Jolla, CA, United States). ANOVA was used to determine significance, and *p* < 0.05 was considered statistically significant.

## Results

### Screening of AI-1 QS Inhibitors

**FIGURE 1:**
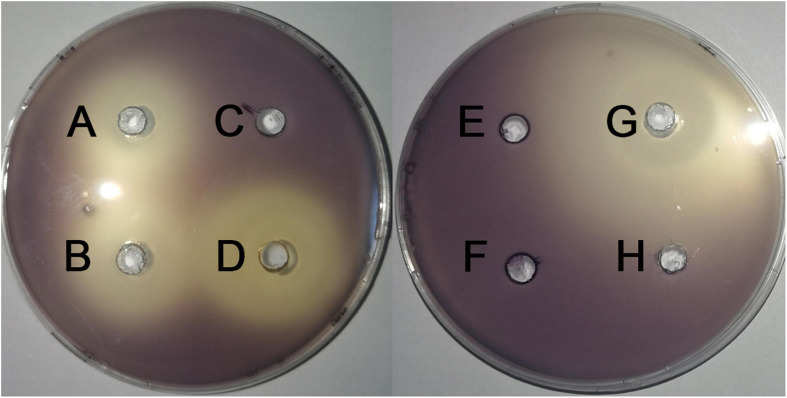
Effects of compounds on the inhibition the violacein production on LB plates. **(A)** paeonol, **(B)** anisic aldehyde, **(C)** polydatin, **(D)** protocatechualdehyde, **(E)** gallate, **(F)** rutin, **(G)** carvacrol, and **(H)** DMSO.

Screening assays, based on QS-mediated violacein production and viability, were performed in broth and agar plates to identify non-bactericidal QS inhibitors (QSIs). Three concentrations of the compound were selected for violacein quantitative assay such that they did not affect the growth of *C. violaceum*. Table 2 shows the effect of test compounds on violacein production in *C. violaceum* 12472, our results indicated that protocatechualdehyde, anisic aldehyde, carvacrol, paeonol, polydatin, saikosaponin A, atractylenolide gallate, veratric acid, and myristic acid inhibited violacein production, and aurantiamarin, baicalin, and rutin promoted violacein production. The other compounds were found to be less effective. A turbid halo around the well on the purple pigmented agar plates was observed for protocatechualdehyde, anisic aldehyde, carvacrol, and paeonol (Table 3 and Figure 1), which indicated a significant inhibition in violacein production without any growth inhibition. Paeonol was selected for further study owing to its excellent anti-QS activity.

**TABLE 2:** Inhibition of *Chromobacterium violaceum* (CV12472) pigments by different compounds.

Test compounds	Selected concentration (μg/mL)	Inhibition rate (%)	Test compounds	Selected concentration (μg/mL)	Inhibition rate (%)
Protocatechuic aldehyde	12.5	16.28 ± 1.85**	Veratric acid	128	5.24 ± 0.37*
	25	27.39 ± 1.60**		256	13.29 ± 2.56**
	50	55.23 ± 5.92**		512	29.76 ± 1.46**
Aniseed aldehyde	12.5	21.41 ± 0.96**	Myristic acid	128	12.93 ± 0.73**
	25	21.56 ± 0.48**		256	12.93 ± 1.46**
	50	31.99 ± 2.22**		512	27.56 ± 3.66**
Paeonol	12.5	18.70 ± 1.96**	Ellagic acid	200	9.27 ± 1.46
	25	23.77 ± 0.79**		400	8.17 ± 6.22
	50	37.81 ± 1.99**		800	15.12 ± 9.51*
	12.5	21.48 ± 1.46**	Glycyrrhiza acid	128	3.78 ± 1.6
Carvacrol	25	31.06 ± 0.31**		256	−4.23 ± 7.25
	50	54.99 ± 0.56**		512	22.27 ± 1.43**
	25	24.81 ± 11.36**	Aurantiamarin	120	−18.94 ± 3.01*
Polydatin	50	10.13 ± 0.44		240	−29.00 ± 9.15**
	100	12.66 ± 2.64*		480	−31.27 ± 10.7**
	6.25	17.41 ± 3.28**	Baicalin	128	−29.74 ± 3.83**
Saikosaponin A	12.5	12.77 ± 3.23**		256	−20.25 ± 2.30**
	25	17.92 ± 1.74**		512	−25.82 ± 3.67**
	128	15.40 ± 3.74**	Rutin	128	−8.22 ± 6.49
Atractylenolide	256	18.79 ± 2.38**		256	−19.80 ± 3.59**
	512	39.86 ± 1.70**		512	−13.32 ± 4.96*
Gallate	64	12.93 ± 1.46**	Puerarin	150	−19.28 ± 14.68*
	128	18.41 ± 4.02**		300	−11.52 ± 3.36
	256	25.73 ± 1.83**		600	0.14 ± 6.27
Calycosin-7-glucoside	128	−0.21 ± 4.80	Sophocarpidine	64	4.88 ± 1.46
	256	11.86 ± 1.78**		128	3.78 ± 1.10
	512	18.25 ± 1.87**		256	−1.71 ± 5.12

*p < 0.05; **p < 0.01 vs. Control. Values represent means ± SD (*n* = 3).

**TABLE 3:** Anti-QS activity of compounds against *C. violaceum* 12472.

Test compounds	Zone of inhibition against *C. violaceum* ATCC 12472 (CV12472) (cm)		
	Total inhibition (d1)	Growth inhibition (d2)	Pigment inhibition (d1–d2)
Paeonol	2.33 ± 0.09	1.19 ± 0.03	1.14 ± 0.12
Aniseed aldehyde	2.53 ± 0.09	0.87 ± 0.09	1.67 ± 0.09
Protocatechuic aldehyde	2.93 ± 0.09	1.47 ± 0.09	1.47 ± 0.09
Carvacrol	5.47 ± 0.19	2.60 ± 0.16	2.87 ± 0.34

*Values represent means ± SD (*n* = 3).*

### *In vitro* Antimicrobial Activity of Paeonol

**FIGURE 2:**
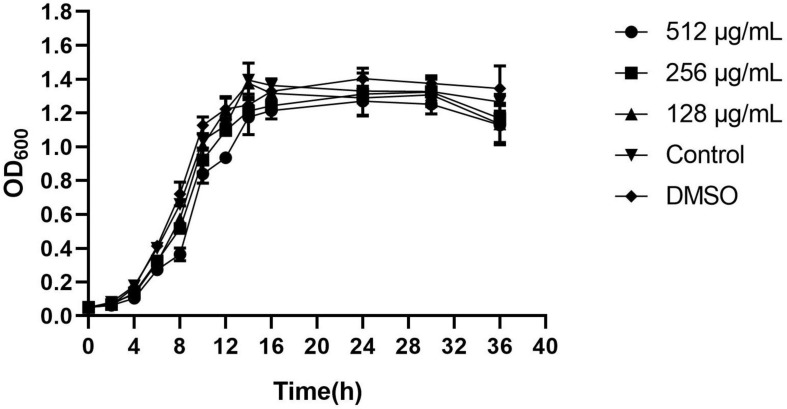
Effects of paeonol on the growth of *Pseudomonas aeruginosa* (PAO1) at different concentrations. All data were expressed as means ± SD (*n* = 3).

The antimicrobial activity of paeonol was evaluated by the MIC microdilution method. Gram negative strains are as follows: *C. violaceum*12472, *E. coli* 25922, *S. typhimurium* 14028, *A. baumannii* 17978, and *P. aeruginosa* PAO1. The MICs of paeonol against these strains were 512, 2,048, 1,024, 512, and 2,048 μg/mL, respectively. Paeonol exhibited an effective anti-bacteria activity against Gram-negative. But no obvious antibacterial effect on MRSA TCH1516. The sub-MIC concentration was selected to evaluate the anti-biofilm and anti-QS activity. Paeonol showed no growth-inhibition activity on *P. aeruginosa* when used at concentrations of 128–512 μg/mL, as determined using the growth curves (Figure 2).

### Anti-biofilm Activity of Paeonol

#### Effect of Paeonol on Biofilm Formation

The results of the inhibitory effects of paeonol on biofilm formation on selected microorganisms are demonstrated in Table 4. Our results indicated that paeonol could decrease the biofilm-forming of Gram-negative bacteria *in vitro*, and the ability in the suppression of biofilm formation was as follows: *C. violaceum* 12472 > *P. aeruginosa* PAO1 > *S. typhimurium* 14028 > *A. baumannii* 17978 > *E. coli* 25922. The sub-MIC concentrations of paeonol were found to have a significant inhibitory effect on the biofilm formation at 36 h. The rates of inhibition also obtained using high concentration of paeonol were 73.75, 74.70, 29.22, 43.32, and 27.18% at 36 h, respectively, compared with the control group. Compared to the effects of selected concentrations on biofilm formation of four *P. aeruginosa* strains, 32.73–74.70, 31.04–67.94, 25.33–30.85, and 57.31–72.52% reduction in biofilm-forming were measured treated with paeonol against PAO1, ATCC 27853, ATCC 9027, and PAO3401, respectively. This study showed that paeonol inhibited the biofilm formation, thus reducing the resistance of bacterials to clinical antibiotics. There was no significant inhibitory effect on biofilm formation against *E. coli* 25922 and MRSA TCH1516. Based on the violacein inhibition and anti-biofilm of the pathogenic bacteria, paeonol was found to be an interesting ingredient against *P. aeruginosa* PAO1.

**TABLE 4:** Effect of sub-MICs of paeonol on inhibition of biofilm formation in bacterial microorganisms.

Bacterial microorganisms	Concentration (μg/mL)	Inhibition rate at 24 h (%)	Inhibition rate at 36 h (%)
*Chromobacterium violaceum* 12472	8	68.84 ± 2.21**	61.83 ± 5.46**
	16	73.10 ± 5.42**	71.33 ± 4.21**
	32	74.75 ± 3.10**	73.75 ± 1.28**
	128	40.43 ± 0.72**	32.73 ± 5.88**
*Pseudomonas aeruginosa* PAO1	256	56.49 ± 2.94**	59.04 ± 6.64**
	512	56.84 ± 3.36**	74.70 ± 4.00**
	128	33.06 ± 15.10**	31.04 ± 17.35**
*Pseudomonas aeruginosa* ATCC 27853	256	40.02 ± 9.06**	44.24 ± 1.99**
	512	46.08 ± 6.62**	67.94 ± 0.49**
	128	24.66 ± 7.88**	25.33 ± 13.31*
*Pseudomonas aeruginosa* ATCC 9027	256	31.79 ± 9.92**	30.27 ± 3.20*
	512	46.73 ± 9.02**	30.85 ± 11.36**
	128	8.34 ± 0.87**	57.31 ± 9.63**
PAO3401	256	22.40 ± 3.14**	67.34 ± 4.86**
	512	33.07 ± 2.93**	72.52 ± 0.84**
	32	36.81 ± 6.65**	23.55 ± 5.06**
*Acinetobacter baumannii* 17978	64	37.68 ± 4.85**	23.40 ± 5.55**
	128	54.08 ± 6.88**	29.22 ± 5.03**
	64	14.85 ± 1.59	24.78 ± 3.86**
*Escherichia coli* 25922	128	17.06 ± 6.89	23.39 ± 11.28**
	256	16.47 ± 7.92	27.18 ± 8.14**
	32	26.36 ± 3.55**	34.06 ± 11.32**
*Salmonella typhimurium* 14028	64	39.17 ± 0.68**	39.54 ± 7.00**
	128	45.27 ± 4.48**	43.32 ± 11.05**
	128	2.40 ± 7.14	10.35 ± 7.96
MRSA TCH1516	256	15.21 ± 8.42	12.11 ± 8.36
	512	26.31 ± 6.03**	17.88 ± 9.68

*P < 0.05; **P < 0.01 vs. Control. Values represent means ± SD (*n* = 3).

#### Effect of Paeonol on Biofilm Development of *P. aeruginosa*

**FIGURE 3:**
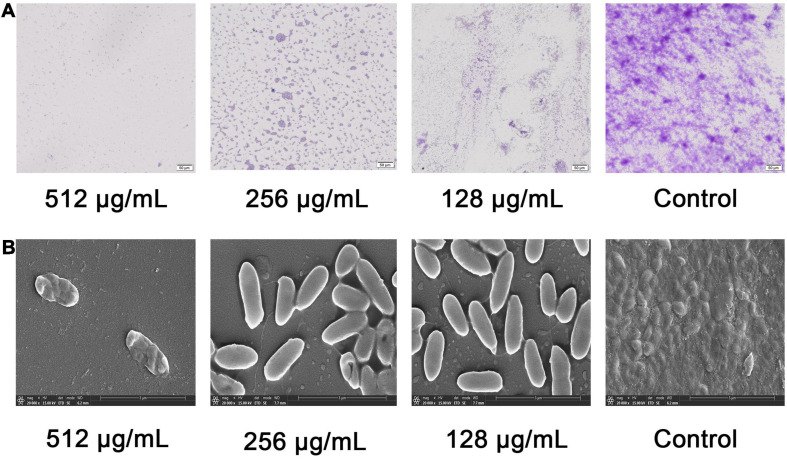
Effects of paeonol on biofilm formation of *P. aeruginosa* (PAO1) at different concentrations. **(A)** Light microscopic images and **(B)** SEM images.

The above anti-biofilm activity was further tested by light microscope and SEM. In the control experiment, the *P. aeruginosa* cells were not treated with the paeonol and a thick biofilm was found. However, compared with the control group, the concentrations of 128,256 and 512 μg/mL paeonol on the glass surface showed a significant reduction in microbial attachment (Figure 3A). The biofilm of *P. aeruginosa* showed a compact surface covered with intact rod-shaped cells. The bacterial cells treated with 128 and 256 μg/mL paeonol also showed normal morphology with clear and complete inner and outer membranes and thin and uniform periplasmic space. But the biofilm is significantly destroyed, which eliminates the basis for pathogenicity (Figure 3B). Based on these results, we concluded that paeonol could effectively inhibit the biofilm formation of *P. aeruginosa* PA01 without interfering its growth in the concentration range of 128–512 μg/mL.

### Effect of Paeonol on Flagellar Motility Against *P. aeruginosa*

**FIGURE 4:**
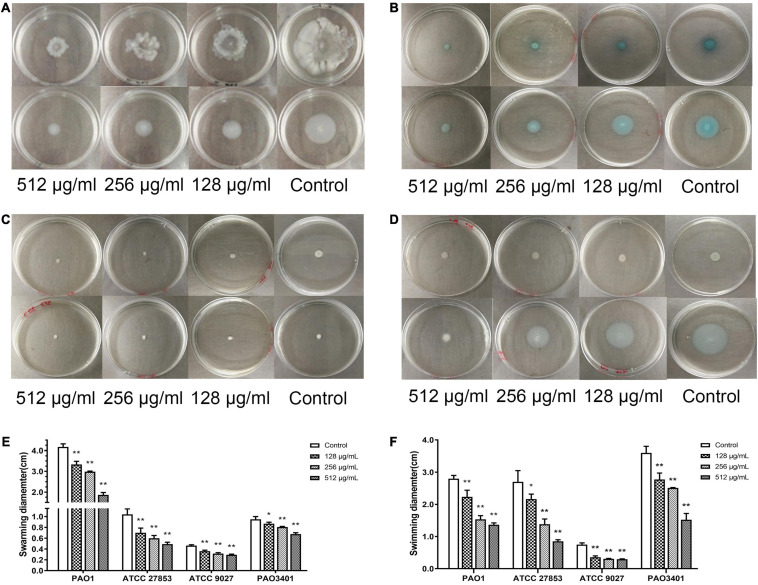
Effect of sub-MICs of paeonol on inhibition of motility in *P. aeruginosa*. *P. aeruginosa* was spotted on a plate supplemented with different concentration paeonol for 24 h by analyzing the swarming motility and swimming motility of PAO1 **(A)**, ATCC 27853 **(B)**, ATCC 9027 **(C)**, and PAO3401 **(D)**, the swarming and swimming diameter statistics **(E,F)**. All data were expressed as means ± SD (*n* = 3). *p < 0.05 and **p < 0.01 versus the control group.

Swarming and swimming motility of *P. aeruginosa* was inhibited by paeonol at concentrations from 128 to 512 μg/mL. An increase in paeonol concentration led to a significant inhibitory effect on *P. aeruginosa* flagellar motility (Figure 4). 55.20, 52.88, 36.39, and 28.77% decrease in swarming motility was determined in the presence of 512 μg/mL paeonol against PAO1, ATCC 27853, ATCC 9027, and PAO3401, respectively. Treatment with paeonol (512 μg/ml) caused significant reduction of swimming motility (51.19, 68.52, 60.71, and 57.69%, respectively) against PAO1, ATCC 27853, ATCC 9027, and PAO3401 (Figures 4A–F). In this study, a dose-dependent decrease was observed in pyocyanin pigment on swarming and swimming motility plates of *P. aeruginosa* ATCC 27853. Interestingly, the outcome of biofilm reduction interrelated definitely with swarming and swimming inhibition, as motility plays a critical role in biofilm adhesion and development.

### Effect of Paeonol on the Virulence Phenotypes of *P. aeruginosa*

#### Pyocyanin

**FIGURE 5:**
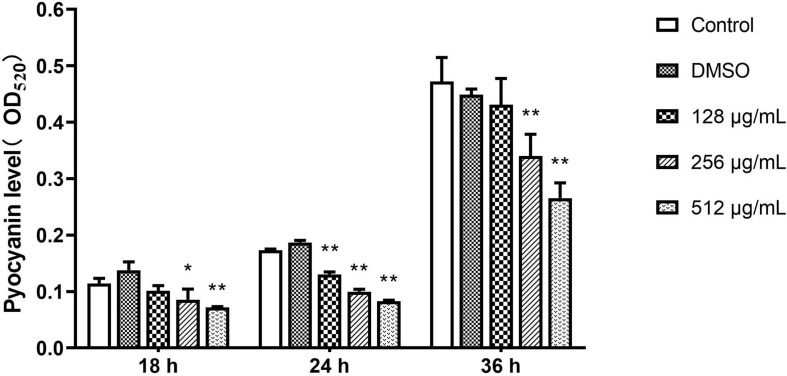
Effect of sub-MICs of paeonol on inhibition of pyocyanin production in *P. aeruginosa* (PAO1). All data were expressed as means ± SD (*n* = 3). *p < 0.05 and **p < 0.01 versus the control group.

The inhibitory effect of paeonol on the production of pyocyanin by *P. aeruginosa* was investigated. Pyocyanin production was significantly decreased by paeonol treatment in a concentration- and time-dependent manner. Paeonol at a concentration of 512 μg/mL was found to have the strongest inhibitory effect on pyocyanin; the rate of inhibition of pyocyanin at 18, 24, and 36 h were 37.19, 52.20, and 43.80%, respectively (Figure 5). Paeonol exhibited relatively weak inhibitory effects on the production of pyocyanin at a concentration of 128 μg/mL.

#### Protease

**FIGURE 6:**
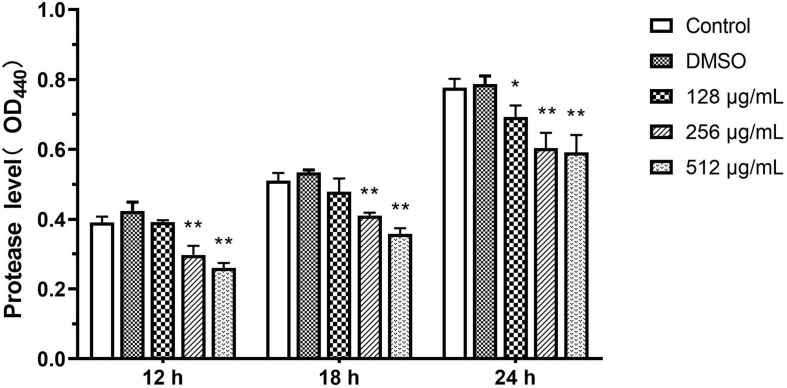
Effect of sub-MICs of paeonol on inhibition of protease activity in *P. aeruginosa* (PAO1). All data were expressed as means ± SD (*n* = 3). *p < 0.05 and **p < 0.01 versus the control group.

The inhibitory ability of paeonol for QS-dependent protease activity was assessed. The extent of protease activity decreased with increasing concentrations of paeonol (128, 256, and 512 μg/mL). Compared with the control group, the rate of inhibition of the proteolytic enzymes with paeonol (512 μg/mL) was determined to be 33.43, 29.80, and 23.85% at 12, 18, and 24 h, respectively, and the inhibitory effect of paeonol was found to decrease over time (Figure 6).

#### Elastase

**FIGURE 7:**
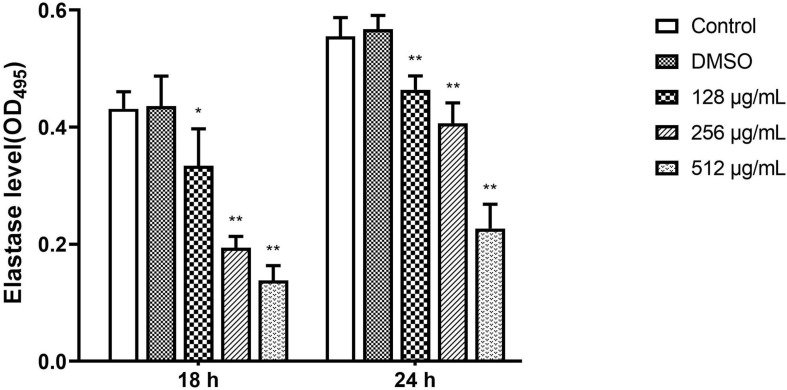
Effect of sub-MICs of paeonol on inhibition of elastase activity in *P. aeruginosa* (PAO1). Elastase activity in *P. aeruginosa* (PAO1) cultured in LB and LB supplemented with different concentration paeonol for 18 h and 24 h were measured by elastin congo red. All data were expressed as means ± SD (*n* = 3). *p < 0.05 and **p < 0.01 versus the control group.

In the elastase activity assay, paeonol significantly inhibited the elastase production of PAO1 on all tested concentrations and the inhibitory effect of paeonol decreased over time. When the concentration of paeonol was 128–512 μg/mL, the elastase activity decreased by 22.59, 55.05, and 68.02% at 18 h, and by 16.48, 26.79, and 59.16% at 24 h, respectively. The results indicated a significantly inhibiting effect of paeonol on elastase activity of *P. aeruginosa* in a concentration- and time-dependent manner (Figure 7).

#### Rhamnolipids

**FIGURE 8:**
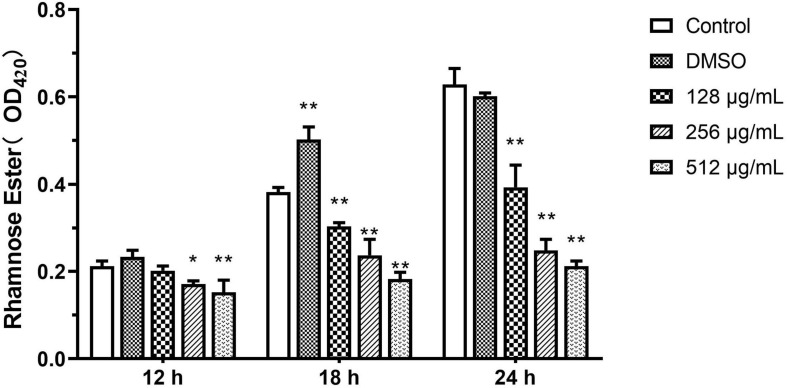
Effect of sub-MICs of paeonol on inhibition of rhamnolipids production in *P. aeruginosa* (PAO1). All data were expressed as means ± SD (*n* = 3). *p < 0.05 and **p < 0.01 versus the control group.

Results from the quantitative analysis indicated that paeonol significantly inhibited rhamnolipid production by PAO1 at all tested concentrations in a concentration-dependent manner. In the presence of 128, 256, and 512 μg/mL paeonol, the levels of rhamnolipids decreased by 37.41, 60.50, and 66.27% at 24 h. At a paeonol concentration of 512 μg/mL, the inhibitory rates of rhamnolipids at 12, 18, and 24 h were 28.20, 52.25, and 66.27%, respectively, suggesting that the paeonol treatment resulted in the strongest inhibitory effect on rhamnolipids at 24 h (Figure 8).

### Effect of Paeonol on AHL Synthesis Activity Against *P. aeruginosa*

**FIGURE 9:**
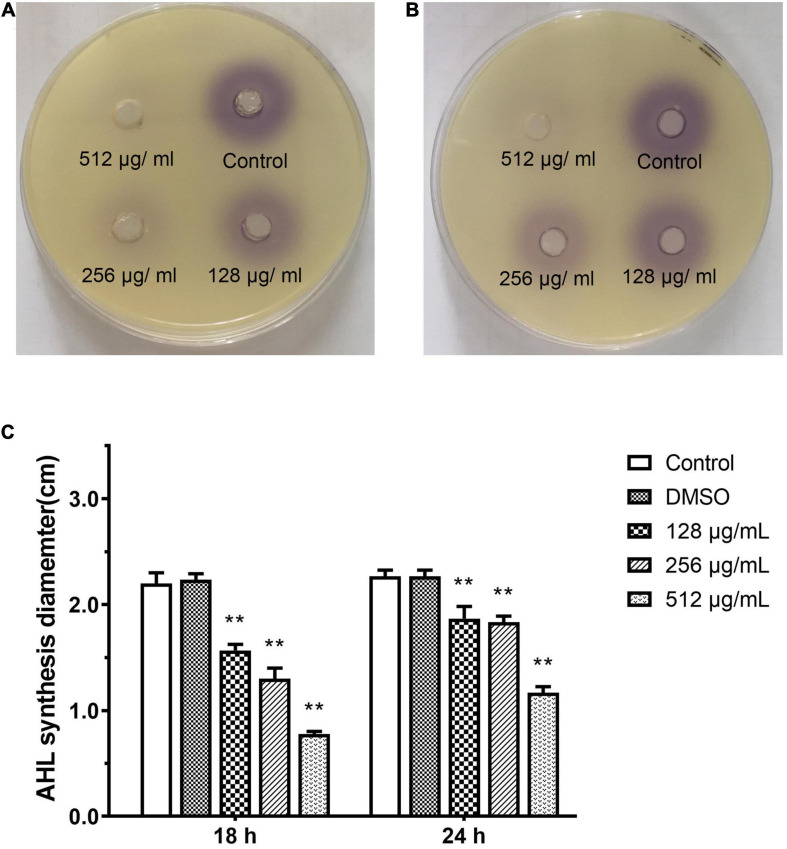
Effect of sub-MICs of paeonol on inhibition of AHL synthesis activity in *P. aeruginosa* (PAO1). Assessment of AHL synthesis activity in *P. aeruginosa* (PAO1) cultured in LB and LB supplemented with different concentration paeonol for 18 h **(A)** and 24 h **(B)** the AHL synthesis diameter statistics **(C)**. All data were expressed as means ± SD (*n* = 3). **p < 0.01 versus the control group.

*N*-acyl L-homoserine lactone-induced violacein from *C. violaceum* 026 was extracted from PAO1 culture that was treated with different concentrations of paeonol. The purple shades of signal molecules at different drug concentrations and various time periods were compared, AHL-mediated production and activity of purple pigment in CV026 were lower in the presence of paeonol, and an obvious inhibition in pigment production was observed upon treatment with paeonol at 18 and 24 h (Figures 9A,B). Quorum sensing signal increased over time was observed, thus, paeonol could effectively inhibit the formation of PAO1 signal molecules in a concentration- and time-dependent manner (Figure 9C). These results indicated paeonol interfered with QS mediated virulence expression of *P. aeruginosa* by reducing the synthesis of AHLs.

### Effect of Paeonol on the Expression of *P. aeruginosa* QS-Regulated Genes

**FIGURE 10:**
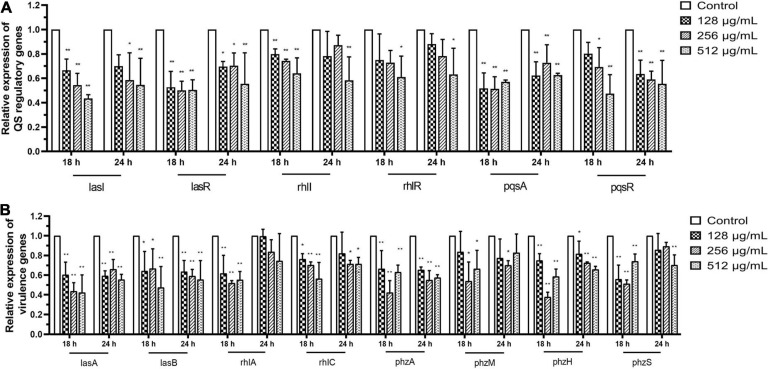
Transcriptional levels of the QS-related **(A)** and virulence genes **(B)** in *P. aeruginosa* with different concentrations paeonol for compared with untreated control at 18 and 24 h were detected by q-PCR. All data were expressed as means ± SD (*n* = 3). *p < 0.05 and **p < 0.01 versus the control group.

Fluorescence real-time PCR was used to determine the expression of QS-regulated genes in *P. aeruginosa* to elucidate the QS-regulating effect of paeonol at the transcriptional level. Our findings indicated that QS-related genes were downregulated. The rates of inhibition in the presence of paeonol (512 μg/mL) at 18 h were as follows: *lasI* 56.60%, *lasR* 49.61%, *rhlI* 35.91%, *rhlR* 38.97%, *pqsA* 42.95%, and *pqsR* 52.65%, *lasA* 57.83%, *lasB* 52.52%, *rhlA* 44.82%, *rhlC* 43.57%, *phzA* 37.01%, *phzM* 33.60%, *phzH* 41.30%, and *phzS* 25.86% (Figures 10A,B). These results indicated that the paeonol-treated group had the strongest inhibitory effect on QS-related gene expression at 18 h, and that the inhibitory effect of paeonol reduced over time. With the increase of the concentration of paeonol, the inhibition of QS target genes (*lasI*, *lasR*, *rhlI*, *rhlR*, *pqsA*, and *pqsR*) and their mediated virulence factors (*lasA*, *lasB*, *rhlA*, *rhlC*, *phzA*, *phzM*, *phzH*, and *phzS*) of *las*, *rhl*, and *pqs* system was enhanced, while 128 μg/mL paeonol resulted in the most significant reduction in *pqs* system at 18 h. The inhibitory effect of paeonol on PAO1 was in concentration- and time-dependent manner, and it may attenuate virulence by interfering with the QS pathway of *P. aeruginosa*.

### Effect of Paeonol on the Survival of *P. aeruginosa* Infected *C. elegans*

**FIGURE 11:**
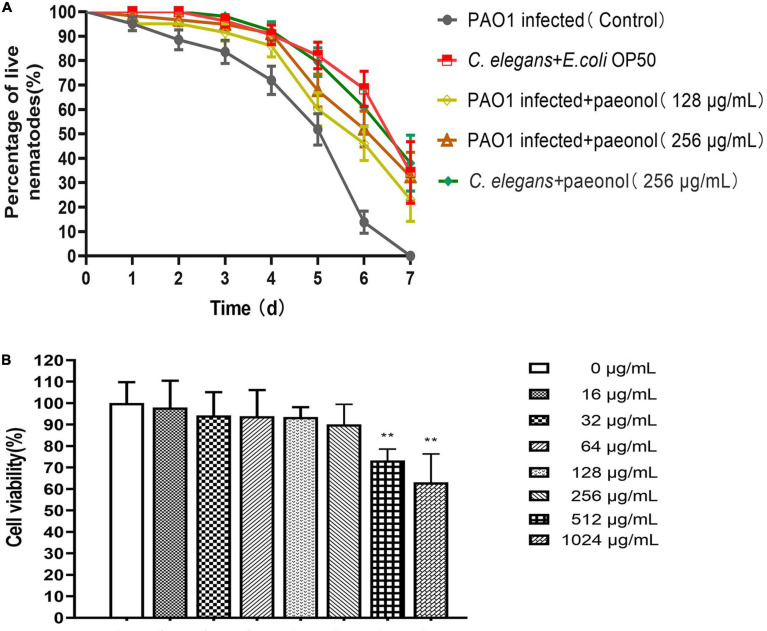
The effect of paeonol on the survival of C. elegans infected with *P. aeruginosa* **(A)**. Effect of different concentrations (0–1,024 μg/mL) of paeonol on IPEC-J2 cell viability activity **(B)**. All data were expressed as means ± SD (*n* = 3). **p < 0.01 versus the control group.

*Pseudomonas aeruginosa* accumulates and produces virulence factors in the gut of *C. elegans* to kill nematodes. The protective effect of paeonol on *P. aeruginosa* infection model of *C. elegans* was further studied. In comparison, *C. elegans* infected by *P. aeruginosa* after treatment with paeonol could significantly improve their survival. In the absence of paeonol, the mortality of *C. elegans* reached 100% after 7 days of PAO1 pathogenicity. The survival rate of *C. elegans* infected with PAO1 were increased about 23.02 and 32.48% when treated by paeonol at the concentrations of 128 and 256 μg/mL. In particular, after 6 days of incubation with paeonol at a concentration of 256 μg/mL, the rate of survival of *C. elegans* N2 was the highest and was found to increase by 38.14% (Figure 11A). Paeonol did not affect the growth of *C. elegans* at the experimental concentrations.

### Paeonol Affect the Viability of IPEC-J2 Cells

For assessing the cytotoxicity of paeonol on IPEC-J2 cells, CCK-8 assay was performed to determine their appearance following treatment with different doses (0, 16, 32, 64, 128, 256, 512, and 1,024 μg/ml) of paeonol for 24 h. Paeonol inhibited the proliferation of cells dose-dependently. The results showed that at the highest concentrations of paeonol (1,024 μg/mL), IPEC-J2 cells viability was 63.08% compared with control. At the lowest concentrations tested (0–256 μg/mL), IPEC-J2 cell viability was unaltered by paeonol (Figure 11B).

## Discussion

Many bacteria sense and communicate with each other through quorum sensing, a gene regulatory mechanism that depends on cell density (Waters and Bassler, 2005). Several naturally occurring compounds have been screened for their anti-QS and anti-biofilm activities in *C. violaceum* (Paczkowski et al., 2017; Zhong et al., 2020). Some potential compounds with anti-QS activity were identified in the preliminary screening, including protocatechualdehyde, anisic aldehyde, carvacrol, and paeonol. Paeonol showed no inhibition in the growth of *C. violaceum* at concentrations of 12.5–50 μg/mL, and production of the AHL-dependent pigment was reduced by nearly 38% at 50 μg/mL, which indicated that paeonol interfered with the QS pathway. Snoussi et al. (2018) have reported that the essential oils and phenolics of *Carum copticum* inhibit violacein production. Similar results also have been reported for the phenolic compounds from plants (Mostafa et al., 2020). There are three QS systems in bacteria: the LuxR/I-type systems, typically used by Gram-negative species, in which the signaling molecule is an *N*-acyl L-homoserine lactone (AI-1); the small oligopeptides (AIP) systems, typically used by Gram-positive species; and the LuxS/furanone metabolites (AI-2) interspecies signaling systems (Galloway et al., 2011; Hawver et al., 2016). Paeonol had shown antibacterial activity against Gram-negative bacteria and interesting disruptive properties against biofilm formation in pathogenic bacterial strains: *C. violaceum*, *P. aeruginosa*, and *A. baumannii*. AHL-mediated quorum sensing is known to play an important role in the formation, development and maturation of biofilms. Therefore, paeonol may penetrate bacterial biofilm interferes with cell signaling and inhibits the QS of most biofilm-related cells. Paeonol reduced the AHL dependent production of violacein and biofilm formation revealed that it interfered with QS systems of Gram-negative pathogens containing various AHL molecules, exhibiting broad-spectrum anti-QS activity. Lv et al. (2020) reported that paeonol could treat *S. typhimurium* infection through the type III secretion system. It was found that paeonol had anti-biofilm-forming activity in *S. typhimurium*, which could be a potential target for anti-infected. Jiao et al. (2016) confirmed that the PDMS surface of paeonol coating showed excellent antibacterial and anticancer activity against Gram-negative and Gram-positive bacteria. The results showed that paeonol could be used as a potential coating material against bacterial biofilm in the industrial environment and on the surface of medical instruments.

*Pseudomonas aeruginosa* is a prevalent hospital-acquired opportunistic pathogen that causes cystic fibrosis. Its pathogenic mechanism is associated with the production of QS-regulated virulence factors and biofilm formation (Sadikot et al., 2005; Ciofu et al., 2015; Azam and Khan, 2019). Compared with that in the untreated PAO1 group, paeonol treatment (128–512 μg/mL) in the experimental group did not result in a direct bactericidal effect, but significantly inhibited the biofilm formation, virulence phenotypes and motility in a concentration- and time-dependent manner. Previous studies have confirmed that phenolic compounds from plants, such as eugenol, tea polyphenols, and thymol, can inhibit the production of virulence-associated factors and biofilm formation in *P. aeruginosa* (Yin et al., 2015; Walsh et al., 2019; Antunes et al., 2021). Scanning electron microscopy results further demonstrated that paeonol decreased biofilm formation against *P. aeruginosa*, possibly interfered with the subsequent steps of surface adhesion or biofilm formation (Pederson et al., 2018; Liu et al., 2019). Additionally, the motility of *P. aeruginosa* is a complex process that is co-regulated by the *las* and *rhl* systems to promote the formation and spread of biofilms (Glessner et al., 1999). *In vitro* studies demonstrated that different concentration of paeonol influenced not only motility of *P. aeruginosa* but also the capability to form biofilm. The results of biofilm formation and flagellar motility demonstrated that paeonol had differences in terms of activity with respect to different strains of *P. aeruginosa*. Our findings indicated that paeonol significantly inhibited the motility of *P. aeruginosa*, suggesting that its inhibitory effect was likely by interfering with QS or by directly acting on the flagella and Type IV pili (Anyan et al., 2014).

Pyocyanin production is controlled by two QS pathways (*rhl* and *pqs*) in *P. aeruginosa* and can interfere with various cellular functions such as chelated iron absorption and respiration (Diggle et al., 2003; Chang et al., 2014). Similarly, paeonol inhibited pyocyanin production in a dose-dependent manner, and the highest inhibition rate was found to be 52.20% at a concentration of 512 μg/mL. Similar inhibitory effects were observed on its QS-regulated gene *rhlI/R* and *pqsA/R*. Protease and elastase are regulated by *lasA* and *lasB* genes, respectively, for the growth and invasion of *P. aeruginosa*, and play an important role in different stages of bacterial colonization and pathogenicity (Husain et al., 2017). With an increase in incubation time, the rate of inhibition of *lasA* protease using 512 μg/mL of paeonol was 23.85–33.43%, whereas that of *lasB* protease was 59.16–68.02%. Rhamnolipids that are synthesized by rhamnolipid synthases *rhlA* and *rhlB* promote the formation and diffusion of biofilms and also kill the polymorphonuclear white blood cells in patients with CF (Bazire and Dufour, 2014). With an increase in culture time, the rate of inhibition of rhamnose achieved using 512 μg/mL of paeonol ranged from 28.20 to 66.27%.

Results from the AHL activity assay showed that the anti-QS activity of paeonol against *P. aeruginosa* may likely result from the binding between AHL and the receptor protein (Smith and Iglewski, 2003; Kaufmann et al., 2006). The QS mechanism on expression at the gene level was further explored, the outcome revealed that paeonol had an obvious inhibitory effect on the QS-related genes, *lasI/R, rhlI/R, pqs/mvfR*, and their mediated virulence factors, *lasA, lasB, rhlA, rhlC, phzA, phzM, phzH, and phzS*. Thus, Paeonol could inhibit the major lactones (C4-HSL, 3-oxo-C12-HSLs, and PQS) that regulated the expression of virulence factors produced by *P. aeruginosa*. These data corroborated with the previous literature where, virulence phenotypes, QS-related genes and biofilm formation of *P. aeruginosa* was descreaced to varying levels by plant-derived compounds and phenolic compounds (Rudrappa and Bais, 2008; Vandeputte et al., 2010; Mok et al., 2020; Rather et al., 2021). The reason may be attributed to the fact that *P. aeruginosa* has a complex QS regulating network, including three main QS circuits, and *las*, the master regulator that induces the expression of both *rhl* and *pqs* (Deziel et al., 2004). The results indicated that paeonol may play a role in multiple targets through QS interference.

Previous research had shown that QS inhibitors block the virulence and biofilm formation of *P. aeruginosa*, protecting *C. elegans* and human lung epithelial cells (O’Loughlin et al., 2013). Therefore, *C. elegans* was used to assess the protective effects of paeonol *in vivo* against *P. aeruginosa* because of the susceptibility to different virulent phenotypes of this bacterium (Tan et al., 1999a; Martin et al., 2017). *P. aeruginosa* accumulates and produces virulence factors, such as pyocyanin, in the intestines of nematodes, which is lethal. Although our research showed that 512 μg/mL paeonol had the most anti-quorum sensing activity on PAO1 *in vitro*, the QS inhibitory concentration is too high when it reaches the clinical treatment level *in vivo*. Therefore, 128–256 μg/ml of paeonol was selected to evaluate the protection of C. elegans infected with PAO1. Paeonol exhibited a significant protective effect at a dose of 256 μg/mL, and the survival rate of the nematodes increased by 38.14% on day 6, shown its anti-infected ability. These results demonstrated paeonol (128–256 μg/mL) could attenuate some virulence factors of *P. aeruginosa* and reduce the pathogenicity of *P. aeruginosa* to improve the survival rate of infected nematodes. In cytotoxicity study, paeonol inhibited IPEC-J2 viability in a dependent manner but had no cytotoxicity in the range of 0–256 μg/mL. Previous studies demonstrated that intragastric LD_50_ value of paeonol in mice is 3,430 mg/kg, which indicated that the toxicity of paeonol is relatively low (Harada and Yamashita, 1969). But the recommended dose of paeonol for the treatment of clinical *P. aeruginosa* infection is 128–256 μg/mL based on the result of viability of IPEC-J2 cells. The anti-virulence mechanism of paeonol against *P. aeruginosa* should be conducted and evaluated if higher doses are more effective *in vivo* by animal experiments in future studies. Its clinical application requires considerable safety and efficacy data.

## Conclusion

In the present study, protocatechualdehyde, anisic aldehyde, carvacrol, and paeonol displayed excellent anti-QS activity. The preliminary screening compounds results provide a platform for exploring QS inhibitors. Taken together, paeonol downregulated the virulence phenotypes by regulating the expression of QS related genes in *P. aeruginosa*. Paeonol attenuated the virulence of *P. aeruginosa* and increased the survival of *C. elegans in vivo*. In addition, paeonol exhibited significant anti-biofilm activity against Gram-negative species including *C. violaceum, P. aeruginosa*, and *A. baumannii*, further demonstrating the potential clinical importance. Hence, paeonol is a promising QSI to be developed a novel and extremely effective anti-virulence and anti-infective drugs. Further research is necessary to elucidate the clinical utility of paeonol against other multi-drug resistance bacteria.

## Data Availability Statement

The raw data supporting the conclusions of this article will be made available by the authors, without undue reservation.

## Author Contributions

DY and HT designed the experimental approach and wrote the manuscript. SH, LZ, FS, GY, YZ, XS, LL, SF, and ZY refined the experimental protocols. DY, SH, and HT performed the animal experiments, physicochemical analyses of particle suspensions. DY prepared the draft. YL and HT made some revision. All authors reviewed and approved the final manuscript.

## Conflict of Interest

The authors declare that the research was conducted in the absence of any commercial or financial relationships that could be construed as a potential conflict of interest.

## Publisher’s Note

All claims expressed in this article are solely those of the authors and do not necessarily represent those of their affiliated organizations, or those of the publisher, the editors and the reviewers. Any product that may be evaluated in this article, or claim that may be made by its manufacturer, is not guaranteed or endorsed by the publisher.
